# Rapidly Containing the First Indigenous Outbreak of Chikungunya in Taiwan—Lessons Learned

**DOI:** 10.3390/tropicalmed6030165

**Published:** 2021-09-10

**Authors:** Ta-Chien Chan, Yu-Fen Hsu, Shao-Chun Huang, Ran-Chou Chen

**Affiliations:** 1 Research Center for Humanities and Social Sciences, Academia Sinica, 128 Academia Road, Section 2, Nankang, Taipei 115, Taiwan; 2 Institute of Public Health, School of Medicine, National Yang Ming Chiao Tung University, No. 155, Section 2, Linong Street, Taipei 112, Taiwan; 3 Department of Health, New Taipei City Government, 192-1, Yingshi Road, Banqiao District, New Taipei City 220, Taiwan; ah4610@ntpc.gov.tw (Y.-F.H.); aj3438@ntpc.gov.tw (S.-C.H.); 4 Department of Biomedical Imaging and Radiological Sciences, National Yang Ming Chiao Tung University, No. 155, Section 2, Linong Street, Taipei 112, Taiwan

**Keywords:** chikungunya virus, outbreak control, ovitrap, diagnosis, GIS (geographic information systems)

## Abstract

The first indigenous outbreak of chikungunya in Taiwan occurred in New Taipei City, northern Taiwan, from August to October 2019. This study identified important containment strategies for controlling the outbreak. The outbreak investigation and ovitrap data were collected from the Department of Health, New Taipei City Government. A geographic information system (GIS) was applied for spatial analysis, and descriptive statistics were used to compute the demographic features and medical visits of confirmed cases. There were 19 residents infected during the outbreak. The source of this outbreak was a mountain trail with abundant *Aedes albopictus*. The atypical symptoms and lack of a rapid test led to multiple clinical visits by the patients (mean: 2.79; standard deviation: 1.65). The clinical symptoms of chikungunya are very similar to those of dengue fever. We noted that only eight patients were polymerase chain reaction (PCR)-positive in their first blood collection, and an average of 3.13 days between illness onset and PCR-positive results. The improved laboratory panel test, targeted and rapid insecticide spraying at the households and their communities, strict closure of the mountain trail, and ovitrap surveillance for evaluating intervention were important approaches to rapidly contain the outbreak.

## 1. Introduction

**Figure 1:**
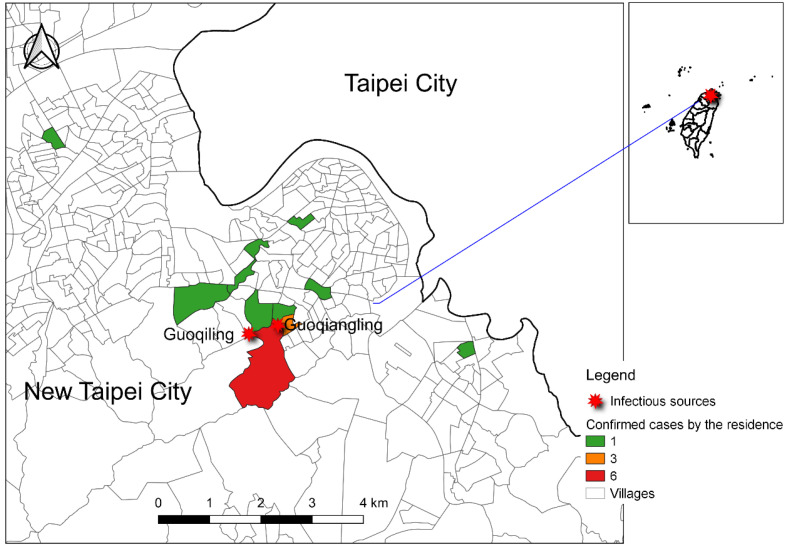
Spatial distribution of 19 confirmed chikungunya cases based on residence and mountain trail infectious sources (from Guoqiling to Guiqiangling) in New Taipei City. The color represents the number of confirmed chikungunya cases in that village. The green color represents one confirmed case in that village, the orange color, three, and the red color, six.

With the rising impact of global warming and intensive international travel among tropical countries, the probability of chikungunya virus (CHIKV) infection in subtropical and temperate countries has been increasing. In Taiwan, the major threatening vector-borne infectious disease in the past two decades was dengue fever, which had large outbreaks in 2002, 2014, and 2015 [1,2]. Imported dengue cases play an important role in the flare-up of local outbreaks [3]. Dengue fever and CHIKV shared the same vector, which is the Aedes mosquitoes [4,5] and their clinical symptoms are quite similar. As with the transmission route of dengue fever, CHIKV was first detected in the imported chikungunya case through fever screening at Taiwan Taoyuan International Airport in November 2006 [6]. From 2007 to 2018, there were, on average, nine imported cases (range: 1–29 cases) annually. In 2019, the number of imported cases reached a peak of 95, originating from Myanmar [7]. The first indigenous outbreak of chikungunya occurred in New Taipei City in northern Taiwan between August and October 2019. There were 19 residents infected during the outbreak (Figure 1). The epidemiological investigation found that they all had a history of activity on the same mountain trail, and the index case lived close to two imported chikungunya cases from Myanmar in July to August 2019. In addition, a phylogenetic tree analysis showed that the viral sequence of the index case was identical to that of an imported case from Myanmar in 2019 [7]. Although enhanced dengue and vector surveillance in communities has been conducted after a large dengue outbreak in 2015 [8], it is important to remain alert to the continuous threat of chikungunya. The enhanced surveillance system from systematic vector surveillance to early case detection was critical for controlling the local outbreak.

The aims of this report were to share the experience of coping with this first CHIKV outbreak based on the past successful experience of controlling dengue fever outbreaks and develop a strategy for the early detection of infected patients.

## 2. Materials and Methods

**Figure 2:**
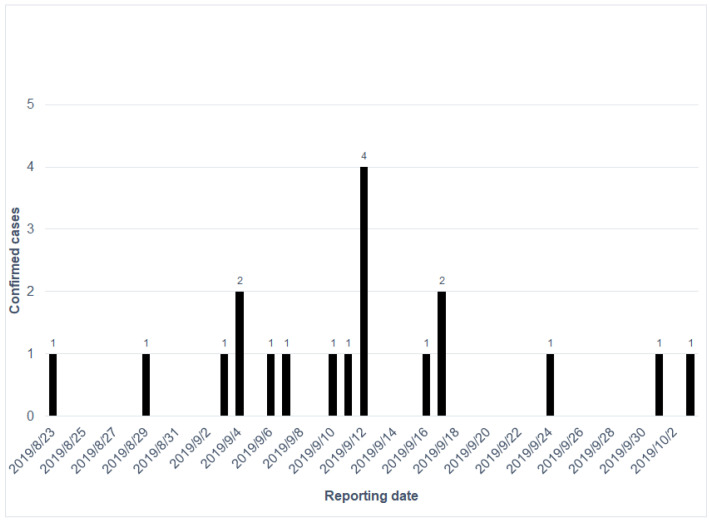
The epicurve of the confirmed chikungunya cases in order of reported dates.

**Figure A1:**
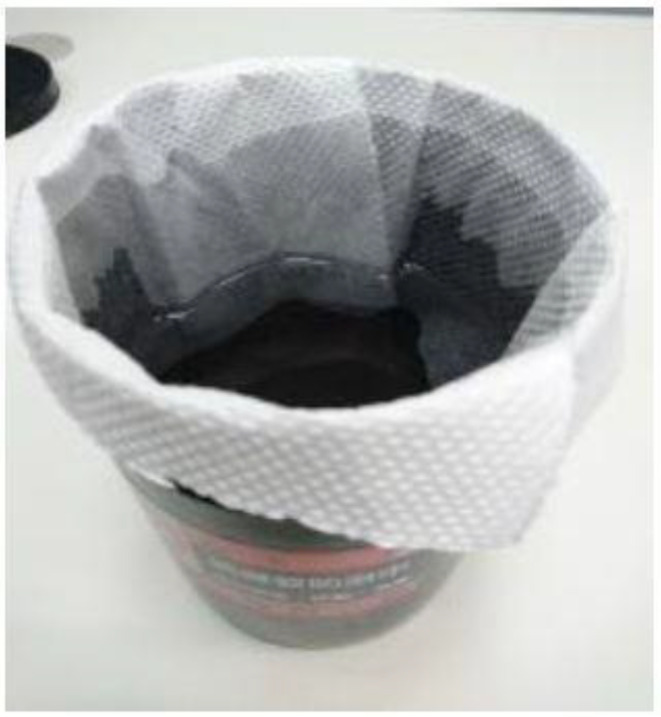
The snapshot of the ovitrap.

During this outbreak, the first CHIKV case was reported on 23 August 2019, and the last CHIKV case on 3 October 2019. Susceptible CHIKV cases were electronically reported by first-line physicians in primary care clinics or hospitals through the National Notifiable Diseases Surveillance System. The epicurve of the confirmed CHIKV cases is shown in Figure 2. We equally divided the duration of the outbreak into two waves by the first 20 days (23 August to 12 September) and the latter 20 days (13 September to 3 October). The number of reported cases peaked on 12 September. An outbreak investigation, ovitrap surveillance, and environmental control were initiated by the local health department. Ovitrap surveillance was deployed in high-risk areas to evaluate the intervention in terms of the reduction in mosquito density. The number of eggs was counted manually each week, and the positive rate was computed as the number of ovitraps with mosquito eggs among deployed ovitraps in the defined area (here, we deployed them in the mountain trail). The protocol for setting up ovitraps was developed by the National Mosquito-Borne Diseases Control Research Center, National Health Research Institute, Taiwan. The ovitrap is a round plastic bucket with a diameter of 12.7 cm (Figure A1 of Appendix A). When we deployed the ovitrap, paper towels and clean water were required to attract the female mosquitoes to hatch the eggs. The insecticide used was *a pyrethroid* insecticide approved by the Taiwan Environmental Protection Administration. We used QGIS 3.4 (QGIS Development Team (2021). QGIS Geographic Information System. Open Source Geospatial Foundation Project. http://qgis.osgeo.org; accessed on 18 August 2021) to visualize the spatial distribution of cases and SPSS 25 (IBM Corp., Armonk, NY, USA) to compute the demographic features of patients; we applied the Mann–Whitney U test to examine their medical visits and the intervals between illness onset and testing results between the two waves.

The temporal resolution of the ovitrap surveillance could not match the waves to the date of case reporting. The date of collecting the first record of the ovitrap was 3 September, and the ovitraps were continuously monitored from 3 weeks after the last few reported cases up until 26 October. Therefore, we classified the first three data points (3, 7, and 14 September) as belonging to the first wave, and the rest of the data points were treated as the second wave (21 and 28 September and 5, 12, 19, and 26 October). We also used the Mann–Whitney U test to compare the eggs and the positive rates between these two waves.

## 3. Results

The clinical symptoms of chikungunya are very similar to those of dengue fever. Of the 19 patients, 17 (89.5%) had joint pain, most often in the small joints of the hands and feet, and 15 (78.9%) had fever of over 38 °C. The subsequent ranking of symptoms included headache (47.4%), rash on the limbs (47.4%), muscle pain (36.8%), and vomiting (31.6%). Thus, the common arthritis- or influenza-like symptoms were diagnosed at first, leading to the patients having multiple clinical visits when the symptoms did not improve.

In Table 1, the average age of the confirmed cases was 58.89 years (standard deviation [SD]: 13.01). Most of the patients (73.7%) were female. The average number of medical visits related to the chikungunya infection was 2.79 (SD: 1.653). The first and last infected cases related to this outbreak were reported on 23 August and 3 October, respectively. In the first wave (23 August to 12 September), the average number of medical visits was 3.15, which was higher than the average of 2.00 in the second wave (13 September to 3 October) (*p* = 0.244). There was an average of 6.54 days between the onset date and reporting date in the first wave, which was higher than the average of 5.50 in the second wave (*p* = 0.521). This should be reduced to less than three days, as with dengue fever outbreaks in Taiwan, to be considered efficient control of the outbreak.

The patients were first tested using the NS1 rapid test for dengue fever, but they were all negative at the beginning. Subsequently, the serological samples were tested using real-time quantitative reverse transcription-polymerase chain reaction (PCR) and immunofluorescent antibody assays. As reported in Table 2, we found that only eight patients were PCR-positive in their first blood collection, and there was an average of 3.13 days between illness onset and PCR-positive results. Five patients were immunoglobulin (Ig) M-positive, with an average of 9.60 days from illness onset to the test results. Four patients tested IgG-positive, with an average of 10.75 days after illness onset.

**Figure 3:**
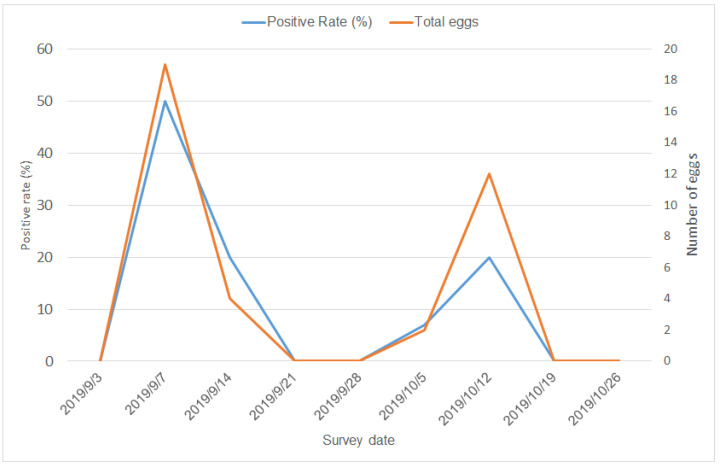
Temporal pattern of positive rates and number of eggs in ovitraps at the mountain trail.

The mountain trail was a natural breeding site for mosquitoes. With intensive insecticide spraying and environmental cleaning, the total number of eggs and the positive rate of ovitraps declined rapidly (Figure 3). In the first wave, the average number of eggs was 7.67 in the first wave and 2.33 in the second waves, respectively (*p* = 0.262), and the average positive rate was 23.33% in the first wave and 4.5% in the second wave (*p* = 0.381). Statistical significance was not attained due to the small sample size.

## 4. Discussion

The lessons learned from this outbreak are as follows and will be beneficial for controlling chikungunya outbreaks in other countries. The clinical symptoms were similar between dengue fever and CHIKV infection. Although first-line clinicians have been alerted to pay attention to dengue fever [9], there is no rapid test for CHIKV to date, which causes a delayed diagnosis. The timeliness of diagnosis can be improved by using a laboratory panel test to differentiate pathogens at the same time. The early case finding was linked to subsequent public health actions to reduce the transmission risk in the communities. Once cases were found in communities, insecticides were immediately sprayed at the infected household on the confirmation date, as well as at the surrounding households within a 50 m buffer. Block-based preventive spraying in the community to reduce potential infected vectors was also effective. The original breeding sources in the mountain trail were also closed until the outbreak was controlled. After the epidemiological investigation of the first few cases, the local health department set up ovitrap surveillance at the mountain trail to evaluate the effectiveness of the intervention. Rapid intervention reduced the risk of chikungunya-infected mosquitoes. In summary, the implementation of rapid insecticide spraying, ovitrap surveillance, closure of the mountain trail, and improved laboratory tests helped contain the outbreak.

The number of asymptomatic chikungunya cases is generally high [10], and the incubation period of chikungunya is shorter than that of dengue fever [11]. Sporadic imported cases cannot be fully detected by screening for fever at an international airport. In the community, reducing the density of mosquitoes and early case detection are important to reduce the chance of subsequent transmission from imported cases. In northern Taiwan, the high population density and its subtropical climate status as a habitat of *Aedes albopictus* [12] will continue to expose the population to a higher risk of vector-borne diseases. 

With the increase in global warming and intensive international travelling, vector-borne diseases have expanded their infected zones from tropical countries to subtropical and temperate countries [13]. For example, Italy suffered the threat of CHIKV in 2007 and 2017 from imported infected cases [14,15]. In many countries, fever screening at the airport is the first checkpoint to block imported cases of CHIKV. However, patients with asymptomatic infection [16] or symptoms without fever are not detected by fever screening. Therefore, a second checkpoint in the communities will be very important. In this study, we demonstrate that ovitrap surveillance and early detection with the enhanced awareness of first-line physicians and panel laboratory testing were crucial for situational awareness of CHIKV. If there was any outbreak of CHIKV, targeted insecticide spraying and the continuous monitoring of ovitrap indicators were able to help control the spread of disease.

The limitation of this study was the small sample size. We could not conduct robust statistical testing and modeling because this outbreak only included 19 patients within a 40-day outbreak. However, the patterns we observed and the control efforts we performed were worth sharing.

In conclusion, the best ways to prevent vector-borne disease outbreaks are still systematic vector surveillance, management of high-risk locations, and shortening of the reporting time for identifying confirmed cases, combined with rapid environmental cleaning and targeted insecticide spraying. Minimizing the gap between surveillance and public health interventions is important to control outbreaks of CHIKV.

## Figures and Tables

**Table 1:** Demographic features of confirmed cases and their medical visits.

Risk Factors	*n*	Mean/Percent	S.D.	Min.	Max.
Age	19	58.89	13.01	34	88
Sex (%)					
Male	5	26.30	-	-	-
Female	14	73.70	-	-	-
# of medical visits	19	2.79	1.65	1	7
Wave 1 (8/23–9/12) ^a^	13	3.15	1.86	*p* = 0.244	
Wave 2 (9/13–10/3) ^a^	6	2.00	0.63		
Days between onset and reporting	19	6.21	5.62	0	23
Wave 1 (8/23–9/12) ^a^	13	6.54	5.95	*p* = 0.521	
Wave 2 (9/13–10/3) ^a^	6	5.50	5.28		

^a^: reporting date.

**Table 2:** Days from illness to serological tests.

Serological Tests	*n*	Days Since Illness Onset			
		Mean	S.D.	Min.	Max.
**First blood collection (*n* = 19)**					
PCR+	8	3.13	2.75	1	9
IgM+	5	9.60	7.73	4	23
IgG+	4	10.75	8.42	4	23
**Second blood collection (*n* = 9)**					
IgM+ and IgG+	9	12.33	4.21	5	20

## Data Availability

The data underlying this article are available in the article and online.

## References

[1] Chang S.F., Huang J.H., Shu P.Y. (2012). Characteristics of dengue epidemics in Taiwan. J. Formos. Med. Assoc..

[2] Yeh C.Y., Chen P.L., Chuang K.T., Shu Y.C., Chien Y.W., Perng G.C., Ko W.C., Ko N.Y. (2017). Symptoms associated with adverse dengue fever prognoses at the time of reporting in the 2015 dengue outbreak in Taiwan. PLoS Negl. Trop. Dis..

[3] Shang C.S., Fang C.T., Liu C.M., Wen T.H., Tsai K.H., King C.C. (2010). The role of imported cases and favorable meteorological conditions in the onset of dengue epidemics. PLoS Negl. Trop. Dis..

[4] Akiner M.M., Demirci B., Babuadze G., Robert V., Schaffner F. (2016). Spread of the invasive mosquitoes aedes aegypti and aedes albopictus in the Black Sea region increases risk of chikungunya, dengue, and zika outbreaks in europe. PLoS Negl. Trop. Dis..

[5] Vairo F., Di Pietrantonj C., Pasqualini C., Mammone A., Lanini S., Nicastri E., Castilletti C., Ferraro F., Di Bari V., Puro V. (2018). The surveillance of chikungunya virus in a temperate climate: Challenges and possible solutions from the experience of Lazio Region, Italy. Viruses.

[6] Shu P.Y., Yang C.F., Su C.L., Chen C.Y., Chang S.F., Tsai K.H., Cheng C.H., Huang J.H. (2008). Two imported chikungunya cases, Taiwan. Emerg. Infect. Dis..

[7] Wei H.Y., Chung Y.J., Chou C.Y., Hung M.L., Tsai Y.F., Tung H.P., Hsieh J.W. (2019). The investigation of the first autochthonous chikungunya outbreak in Taiwan, 2019. Taiwan Epidemiol. Bull..

[8] Wang W.H., Lin C.Y., Chang K., Urbina A.N., Assavalapsakul W., Thitithanyanont A., Lu P.L., Chen Y.H., Wang S.F. (2019). A clinical and epidemiological survey of the largest dengue outbreak in Southern Taiwan in 2015. Int. J. Infect. Dis..

[9] Kao J.H., Chen C.D., Tiger Li Z.R., Chan T.C., Tung T.H., Chu Y.H., Cheng H.Y., Liu J.W., Shih F.Y., Shu P.Y. (2016). The critical role of early dengue surveillance and limitations of clinical reporting—Implications for non-endemic countries. PLoS ONE.

[10] Nakkhara P., Chongsuvivatwong V., Thammapalo S. (2013). Risk factors for symptomatic and asymptomatic chikungunya infection. Trans. R. Soc. Trop. Med. Hyg..

[11] Rudolph K.E., Lessler J., Moloney R.M., Kmush B., Cummings D.A. (2014). Incubation periods of mosquito-borne viral infections: A systematic review. Am. J. Trop. Med. Hyg..

[12] Chen W.J. (2018). Dengue outbreaks and the geographic distribution of dengue vectors in Taiwan: A 20-year epidemiological analysis. Biomed. J..

[13] Ogden N.H. (2017). Climate change and vector-borne diseases of public health significance. FEMS Microbiol. Lett..

[14] Rezza G., Nicoletti L., Angelini R., Romi R., Finarelli A.C., Panning M., Cordioli P., Fortuna C., Boros S., Magurano F. (2007). Infection with chikungunya virus in Italy: An outbreak in a temperate region. Lancet.

[15] Venturi G., Di Luca M., Fortuna C., Remoli M.E., Riccardo F., Severini F., Toma L., Del Manso M., Benedetti E., Caporali M.G. (2017). Detection of a chikungunya outbreak in Central Italy, August to September 2017. Euro Surveill..

[16] Bustos Carrillo F., Collado D., Sanchez N., Ojeda S., Lopez Mercado B., Burger-Calderon R., Gresh L., Gordon A., Balmaseda A., Kuan G. (2019). Epidemiological evidence for lineage-specific differences in the risk of inapparent chikungunya virus infection. J. Virol..

